# Prosocial Gains and Losses: Modulations of Human Social Decision-Making by Loss-Gain Context

**DOI:** 10.3389/fpsyg.2021.755910

**Published:** 2021-10-28

**Authors:** Chunliang Feng, Yijie Zhang, Zhixin Zhang, Jie Yuan

**Affiliations:** ^1^Key Laboratory of Brain, Cognition and Education Sciences (South China Normal University), Ministry of Education, Guangzhou, China; ^2^School of Psychology, South China Normal University, Guangzhou, China; ^3^Center for Studies of Psychological Application, South China Normal University, Guangzhou, China; ^4^Guangdong Key Laboratory of Mental Health and Cognitive Science, South China Normal University, Guangzhou, China

**Keywords:** loss aversion, social decision making, dual-process framework, prospect theory, do-no-harm principle

## Abstract

The role of the loss-gain context in human social decision-making remains heavily debated, with mixed evidence showing that losses (vs. gains) boost both selfish and prosocial motivations. Herein, we propose that the loss context, compared to the gain context, exacerbates intuitive reactions in response to the conflict between self-interest and prosocial preferences, regardless of whether those dominant responses are selfish or altruistic. We then synthesize evidence from three lines of research to support the account, which indicates that losses may either enhance or inhibit altruistic behaviors depending on the dominant responses in the employed interactive economic games, prosocial/proself traits, and the explicit engagement of deliberative processes. The current perspective contributes to the ongoing debate on the association between loss-gain context and human prosociality by putting forward a theoretical framework to integrate previous conflicting perspectives.

## Introduction

Human behaviors and cognitions are disproportionately influenced by negative events relative to positive or neutral events, demonstrating a “negativity bias” (Baumeister et al., 2001; Rozin and Royzman, 2001; Vaish et al., 2008). Negative events are more preferentially attended to (Hansen and Hansen, 1988; Cooper and Langton, 2006), difficultly disengaged from (Salemink et al., 2007), and heavily weighted (Skowronski and Carlston, 1989) compared to other events. For instance, task-irrelevant negative events often induce a larger interference on an individual’s ongoing task performance than that induced by irrelevant neutral or positive events (Williams et al., 1996; Feng et al., 2018). Notably, the priority in the processing of negative information occurs at early processing stages (Huang and Luo, 2006; Olofsson et al., 2008; Kappenman et al., 2015), operates automatically, and is independent of the information load and awareness (Hansen and Hansen, 1988; Öhman, 2005). Negativity bias represents one of the most basic and overarching psychological principles (Baumeister et al., 2001; Rozin and Royzman, 2001) and might serve the evolutionarily adaptive function of avoiding detrimental situations. Herein, we focus on how negativity bias manifests in human decision-making, especially in social contexts.

## Effects of Loss on Individual Decision-Making

In accord with the asymmetrical effects of the bad and the good in other domains, it has been proposed that losses exert a stronger effect on human judgments and decisions than comparable gains (Hilbig, 2009; Neumann and Böckenholt, 2014; Mrkva et al., 2020; Brown et al., 2021). For instance, many studies have identified that the behavioral avoidance of choices leads to potential losses, even when accompanied by equal-sized or substantially larger gains (Tversky and Kahneman, 1991; Novemsky and Kahneman, 2005; Kahneman and Tversky, 1979). In contrast, other studies have not found this effect, particularly for small to medium losses (see review in Yechiam and Hochman, 2013; Gal and Rucker, 2018). Notwithstanding, studies have shown that even small losses increase arousal, performance, and cortical processing (see review in Yechiam and Hochman, 2013). The asymmetric effects of losses on human behaviors have been historically attributed to higher subjective weighting of losses than gains in prospect theory, i.e., loss aversion (Kahneman and Tversky, 1979). However, more recent empirical evidence has indicated that losses might only induce global increases in sensitivity to task reinforcements, which do not necessarily give rise to loss aversion at the behavioral level (Hochman and Yechiam, 2011; Yechiam and Telpaz, 2011, 2013; Yechiam and Hochman, 2013).

The effect of the loss-gain context on human decision-making could be understood in the dual-process framework (Epstein, 1994; Kahneman, 2011; Feng et al., 2015; Loewenstein et al., 2015); the intuitive system (i.e., System 1) consists of affective processes that are fast and automatic, while the deliberative system (i.e., System 2) includes rational processes that are slower, more analytical and calculating in nature. In particular, it has been increasingly acknowledged that asymmetry in the impact of gains and losses reflects the essential role of intuitive emotional processes in human decision-making (e.g., Slovic et al., 2007; Loewenstein et al., 2015). For instance, the modulation of loss context on decision-making engages the involvement of the amygdala as a key node for emotional processing (De Martino et al., 2006; Roiser et al., 2009; Canessa et al., 2013), the activity of which predicts individual differences in the susceptibility to loss context (Roiser et al., 2009). Moreover, the loss-gain asymmetry in risky decisions is significantly attenuated among amygdala-lesioned patients compared with healthy controls, who typically exhibit higher sensitivity to losses than gains (De Martino et al., 2010). Last, experimental manipulations that facilitate deliberative processes, such as intentional emotional regulation strategy and presenting choices in a foreign language, effectively decrease the loss-elicited bias in decision-making (Sokol-Hessner et al., 2009, 2013; Keysar et al., 2012), as well as associated physiological arousal (Sokol-Hessner et al., 2009; Sheng et al., 2020) and amygdala activations (Sokol-Hessner et al., 2013) in response to losses. In contrast, experimental procedures that promote intuitive processes (e.g., incidental fear cues) enhance susceptibility to loss context as well as amygdala responses or skin conductance responses to losses (Stancak et al., 2015; Schulreich et al., 2016, 2020).

Together, previous findings have indicated that human decisions pertaining to losses and gains are quite different from each other, and loss-gain asymmetry is closely related to the intuitive emotional system. Although the past decades have witnessed a wealth of empirical evidence on the influence of loss context on human individual decision-making under risk and uncertainty (Neumann and Böckenholt, 2014), it remains controversial how loss-gain context modulates human social behaviors or preferences.

## Evidence That Losses (Vs. Gains) Decrease Prosociality

A large body of evidence has indicated that individuals exhibit lower levels of prosocial behaviors in the loss context than in the gain context. In particular, some studies have indicated that proposers in the dictator or ultimatum game show a lower dislike for advantageous inequality when outcomes are framed as losses than when outcomes are framed as gains (Lusk and Hudson, 2010; Neumann et al., 2018; Fiedler and Hillenbrand, 2020). Moreover, across die-under-the-cup and coin-toss tasks, people are more motivated to cheat to avoid loss than to make gains of identical size (Van Yperen et al., 2011; Grolleau et al., 2014, 2016; Schindler and Pfattheicher, 2017; Sun et al., 2017; Markiewicz and Czupryna, 2020; Markiewicz and Gawryluk, 2020). Last, individuals are more likely to approve of obtaining “insider information” in response to hypothetical scenarios (Kern and Chugh, 2009) and more prone to making self-serving mistakes in a die-roll task (Leib et al., 2019) in the loss context than in the gain context.

These findings align with prospect theory, which holds that people dislike losses more than they like equivalent gains, that is, loss aversion (Kahneman and Tversky, 1979). In other words, it is psychologically more aversive to endure a loss than to give up an equivalent gain to enhance the net payoff of others (Reinders Folmer and De Cremer, 2012). According to this account, it is likely that the effect of the loss context on human prosociality is mediated by the intuitive emotional processes, given the critical role of emotional processing in loss aversion (Sokol-Hessner and Rutledge, 2019). Alternatively, these findings could be accounted for by a recent attention-allocation model positing that losses increase attention to the task, which in turn facilitates the reaction to the reinforcement structure (Yechiam and Hochman, 2013). That is, losses might enhance the motives to maximize self-interest, in line with previous findings that losses lead to greater maximization during individual decision-making (Bereby-Meyer and Erev, 1998; Yechiam and Ert, 2007).

## Evidence That Losses (Vs. Gains) Increase Prosociality

Many other studies have demonstrated that people exhibit enhanced concern for the welfare of others and for social norms in the loss domain than in the gain domain. Across several countries, ultimatum bargaining over losses induces higher demands from responders and higher offers from proposers than ultimatum bargaining over gains, which suggests that fairness is assigned a higher weight in the loss domain than in the gain domain (Buchan et al., 2005; Zhou and Wu, 2011; Baquero et al., 2013; Guo et al., 2013; Wu et al., 2014; Neumann et al., 2017). Likewise, fairness is more accessible, fairness norms are stronger, and resource allocation is more equal when bargaining over losses than over gains. Notably, the effect of the loss context on allocation behavior is mediated by enhanced fairness motivations and attenuated self-interest motives; that is, fairness concerns dominate over self-interest in the loss context (Leliveld et al., 2009). Moreover, people who act as dictators in the dictator game are intrinsically motivated to share more money with recipients in a loss domain than in a gain domain, thereby demonstrating a higher level of generosity in the loss context (Baquero et al., 2013; Yin et al., 2017; Thunström, 2019; Cochard et al., 2020). Last, people are less likely to harm others by exclusion (van Beest et al., 2003, 2005) and are more cooperative (De Dreu et al., 1992) in the loss context than in the gain context.

These findings are consistent with the “do-no-harm” principle asserting that people are unwilling to harm others to benefit themselves (Baron, 1995; Van Beest et al., 2003, 2005). Therefore, it is likely that losses are more readily appraised as a kind of harm to others than gains, which in turn enhance people’s concern for others through aversion to imposing harm on them (Leliveld et al., 2009; Thunström, 2019). This account is consistent with the evidence showing that the enhancement of human altruism by the loss context is mediated by the relative importance between self-reported proself and prosocial concerns (Van Beest et al., 2005; Leliveld et al., 2009). Notably, the “do-no-harm” principle is implemented as more heuristics than analytics, such that people’s decisions according to this principle are often different from their reasoning about available options (Ritov and Baron, 1992; Baron, 1995). Therefore, this account also emphasizes that the modulation of the loss context on human prosociality relies on the operation of intuitive heuristics rather than a deliberate, controlled reasoning process.

## Discussion

The current literature has provided seemingly contradictory evidence indicating that loss context (compared to gain context) could facilitate both self-interest concerns and prosocial motivations. A plausible account for the current mixed evidence is that the loss context promotes reflexive or automatic responses regardless of whether they are selfish or prosocial. In other words, self-interest concerns might dominate over prosocial concerns in some contexts or for some people, whereas altruistic reactions represent intuitive responses for others, and those intuitive reactions are further amplified by the loss context. This conjecture, which provides a potential reconciliation for the apparently discrepant relationship between losses and prosociality in the current literature, has been supported by several lines of research.

First, previous studies have revealed that loss contexts often enhance human generosity (Baquero et al., 2013; Yin et al., 2017; Thunström, 2019; Cochard et al., 2020) but usually reduce human honesty (Van Yperen et al., 2011; Grolleau et al., 2014, 2016; Schindler and Pfattheicher, 2017; Sun et al., 2017; Markiewicz and Czupryna, 2020; Markiewicz and Gawryluk, 2020). The opposite effects of losses on the two distinct social preferences could be attributed to the reason that human generosity mainly reflects intuitive responses, whereas human honesty often requires cognitive control. On the one hand, generous decisions, such as voluntary giving and charitable donations, consistently engage emotion-related regions (e.g., ventral striatum) rather than brain regions implicated in cognitive control [e.g., lateral prefrontal cortex (lPFC); Moll et al., 2006; Harbaugh et al., 2007; Zaki and Mitchell, 2011; Ty et al., 2017], which is thought to reflect the intrinsic value of generosity (Zaki and Mitchell, 2013). Moreover, excitatory stimulation of the lPFC decreases voluntary giving (Ruff et al., 2013), whereas inhibitory stimulation of the lPFC leads to increases in generosity (Ruff et al., 2013; Christov-Moore et al., 2017; Yin et al., 2017). Last, some studies have found that people might exhibit an enhanced level of generosity under cognitive load (Schulz et al., 2014) or time pressure (Cappelletti et al., 2011), which constrain cognitive control; however, it should be noted that recent meta-analyses indicate that the effects of cognitive manipulations are modulated by gender (Rand et al., 2016) or nonsignificant (Fromell et al., 2020). On the other hand, costly honest decisions involve cognitive control regions (Abe and Greene, 2014; Yin et al., 2016) as well as stronger functional coupling between these regions (Dogan et al., 2016). In addition, lesions to the lPFC significantly reduce honesty behaviors (Zhu et al., 2014), and excitatory stimulation of the lPFC leads to dramatic increases in honesty (Maréchal et al., 2017). Last, restraining people’s deliberate thinking decreases costly honesty (Mead et al., 2009; Gino et al., 2011; Shalvi et al., 2012). In light of these findings, the opposite effects of loss context on human generosity and honesty are consistent with the account asserting that losses promote intuitive reactions during social decision-making.

Second, several studies have demonstrated that whether losses enhance selfish or altruistic behaviors depends on individual variations in social value orientations, such that loss contexts promote prosocial individuals’ altruistic preferences but curtail individualists’ altruistic concerns (De Dreu and McCusker, 1997; Reinders Folmer and De Cremer, 2012). For instance, prosocial individuals cooperate more in a loss domain than in a gain domain, whereas proself individuals cooperate less in a loss domain than in a gain domain (De Dreu and McCusker, 1997). In the same vein, females are generally more generous than males (My et al., 2018; Cochard et al., 2020); accordingly, it has been reported that females are more generous in the loss domain in than the gain domain, but males do not exhibit significant differences in generosity between domains (Cochard et al., 2020). These findings agree with the idea that the effect of losses on human prosociality depends on distinct intuitive (selfish or prosocial) responses exhibited by different individuals.

Third, the effect of losses on human altruism is attenuated by the engagement of the deliberative system. For instance, providing participants with more time to consider the outcomes associated with different options decreased the effect of the loss context (Zhou and Wu, 2011). Moreover, the effect of the loss context on human generosity significantly decreases when individuals are encouraged to engage in rational behaviors by reminding them of the financial consequences of their decisions (Thunström, 2019). These findings complement two other lines of research showing that (i) a loss situation can accelerate the consumption of self-control resources (Liu et al., 2020) and that (ii) individuals are less rational in the loss domain (Baron, 1995), such that people are less likely to harm a group in a loss domain than in a gain domain to achieve a better overall outcome (Van Beest et al., 2005). These findings indicate that the modulation of losses on human prosociality depends on reflexive processes, whereas engagement in more deliberative mental processes can attenuate the effect of losses.

Taken together, there is a growing body of empirical evidence supporting the idea that the modulation of loss context on human prosociality might be achieved by magnifying intuitive responses in the task at hand, independent of whether these relatively automatic reactions are altruistic or selfish. This account provides a promising reconciliation for the apparently mixed evidence on the relationship between losses and human prosocial preferences.

## Limitations and Future Directions

Several limitations and corresponding future directions should be noted. First, it is important for future studies to reveal the nature of intuitive responses of the employed tasks. For instance, it has been revealed that honesty could be automatic or controlled depending on the types of tasks utilized (Köbis et al., 2019). Relatedly, future studies need to characterize participants in terms of their personality traits associated with prosociality and/or negativity (e.g., Brunell et al., 2014; Buelow and Brunell, 2020). On the one hand, some people are more altruistic than others, which in turn likely leads to different intuitive responses among individuals (Rand et al., 2016). On the other hand, personality traits such as neuroticism/narcissism modulate one’s reactivity to negative events (Canli et al., 2001; Chan et al., 2007); it is plausible that the more individuals are sensitive to negativity, the more likely their prosocial behaviors are modulated by loss contexts. The heterogeneity in tasks and/or samples utilized in the literature might explain the null effects of the loss-gain context on human prosociality reported in several studies (e.g., Antinyan, 2014).

Second, although the evidence synthesized in the Discussion section aligns with the hypothesis proposed in the current perspective, these studies did not directly test the hypothesis. As mentioned above, future studies need to better control for intuitive responses of employed tasks and recruited samples to directly test the hypothesis.

Third, in light of the attention-allocation model, an alternative account of inconsistent findings on losses and prosociality could be that losses increase people’s attention to the task, which in turn makes people act less noisily but instead more in line with the dominant prosocial or proself response (Hochman and Yechiam, 2011; Yechiam and Hochman, 2013). Although the attention-allocation model has considered attention as a component of the deliberative system (e.g., Yechiam and Hochman, 2013), many brain imaging studies has indicated that attention bias to negative events is automatic and thus associated with brain activity in the intuitive system (e.g., amygdala; Dolan and Vuilleumier, 2003; Albert et al., 2017) or occurs at early processing stages (Olofsson et al., 2008; Luo et al., 2010). Therefore, attention might mediate the promotion of losses on intuitive reactions, in line with the hypothesis proposed in the current perspective. This conjecture is consistent with several lines of research. First, the effects of losses on generosity could be significantly diminished by the engagement of the deliberative system (Thunström, 2019). Second, many studies have indicated that optimizing one’s performance (or decreasing the randomness of behavior) does not necessarily rely on controlled processes but could be closely related to emotional intuitive processes (Bechara and Damasio, 2005; Poppa and Bechara, 2018). Nevertheless, future studies could directly test these alternative hypotheses by employing brain imaging techniques.

## Conclusion

It has been well demonstrated that people treat losses and gains differently during individual decision-making, which is closely related to differential emotional responses to losses and gains (Ashraf et al., 2005; Camerer, 2005). However, it remains unclear how human social decision-making might be different across loss and gain domains, with mixed evidence showing that losses boost both selfish and prosocial motivations. From the current perspective, we aimed to propose a potential account to reconcile previous seemingly inconsistent findings, arguing that the modulation of losses on human social decision-making relies on intuitive emotional processes, similar to the role of losses in individual decision-making. We then synthesized evidence from three lines of research to support the account, revealing that losses may either increase or curtail selfish behaviors depending on dominant responses in the employed interactive economic games, prosocial/proself traits, and the explicit engagement of deliberative processes.

The current perspective contributes to the ongoing debate on the association between loss-gain context and human prosociality by enabling a theoretical framework to integrate contradictory perspectives in the literature. Moreover, the context- and person-dependent effect of losses may have significant practical implications pertaining to the understanding of human altruism in the downturn as well as the design of institutions to facilitate social preferences, emphasizing that loss context might enhance social preferences in some contexts and for some people but may have unintended side effects for others.

## Data Availability Statement

All datasets generated for this study are included in the manuscript.

## Author Contributions

CF wrote the manuscript. YZ, ZZ, and JY provided important comments to improve the manuscript. All authors contributed to the article and approved the submitted version.

## Funding

This study was supported by the National Natural Science Foundation of China (31900757 and 32020103008) and Natural Science Foundation of Guangdong Province (2021A1515010746).

## Conflict of Interest

The authors declare that the research was conducted in the absence of any commercial or financial relationships that could be construed as a potential conflict of interest.

## Publisher’s Note

All claims expressed in this article are solely those of the authors and do not necessarily represent those of their affiliated organizations, or those of the publisher, the editors and the reviewers. Any product that may be evaluated in this article, or claim that may be made by its manufacturer, is not guaranteed or endorsed by the publisher.
